# Proteomics and Post-Translational Modifications of Starch Biosynthesis-Related Proteins in Developing Seeds of Rice

**DOI:** 10.3390/ijms22115901

**Published:** 2021-05-31

**Authors:** Piengtawan Tappiban, Yining Ying, Feifei Xu, Jinsong Bao

**Affiliations:** 1 Key Laboratory of Nuclear Agricultural Sciences of Ministry of Agriculture and Zhejiang Province, Institute of Nuclear Agricultural Sciences, College of Agriculture and Biotechnology, Zhejiang University, Zijingang Campus, Hangzhou 310058, China; Piengtawan.tap@hotmail.com (P.T.); ying_erin@163.com (Y.Y.); xuxufei@zju.edu.cn (F.X.); 2 Hainan Institute of Zhejiang University, Yazhou Bay Science and Technology City, Yazhou District, Sanya 572025, China

**Keywords:** rice, starch biosynthesis, proteomics, posttranslational modification, starch functionality, cooking and eating quality

## Abstract

Rice (*Oryza sativa* L.) is a foremost staple food for approximately half the world’s population. The components of rice starch, amylose, and amylopectin are synthesized by a series of enzymes, which are responsible for rice starch properties and functionality, and then affect rice cooking and eating quality. Recently, proteomics technology has been applied to the establishment of the differentially expressed starch biosynthesis-related proteins and the identification of posttranslational modifications (PTMs) target starch biosynthesis proteins as well. It is necessary to summarize the recent studies in proteomics and PTMs in rice endosperm to deepen our understanding of starch biosynthesis protein expression and regulation, which will provide useful information to rice breeding programs and industrial starch applications. The review provides a comprehensive summary of proteins and PTMs involved in starch biosynthesis based on proteomic studies of rice developing seeds. Starch biosynthesis proteins in rice seeds were differentially expressed in the developing seeds at different developmental stages. All the proteins involving in starch biosynthesis were identified using proteomics methods. Most starch biosynthesis-related proteins are basically increased at 6–20 days after flowering (DAF) and decreased upon the high-temperature conditions. A total of 10, 14, 2, 17, and 7 starch biosynthesis related proteins were identified to be targeted by phosphorylation, lysine acetylation, succinylation, lysine 2-hydroxyisobutyrylation, and malonylation, respectively. The phosphoglucomutase is commonly targeted by five PTMs types. Research on the function of phosphorylation in multiple enzyme complex formation in endosperm starch biosynthesis is underway, while the functions of other PTMs in starch biosynthesis are necessary to be conducted in the near future.

## 1. Introduction

**Figure 1:**
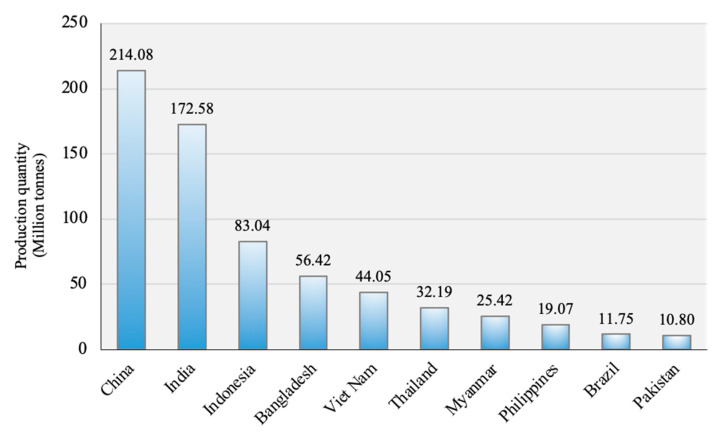
Production quantity of rice in 2018 from the top 10 countries based on FAOSTAT [1].

Rice (*Oryza sativa* L.) is one of the most consumed cereal grains for half of the world’s population and ranks as the third-largest crop, after sugarcane and maize [1,2]. The production quantity of rice was an estimated 214.08 million tonnes reported in 2018 [1], of which China, India, and Indonesia were the top-three highest production countries (Figure 1). Cultivated rice consists of two subspecies, *O. sativa japonica* and *O. sativa indica* [3,4], which are highly distinct in terms of geographical distribution, visible morphological traits [5], and physiological characteristics (e.g., biotic and abiotic stress responses, cold tolerance, and seed quality) [6].

Rice seed consists of a minuscule embryo containing most of the genetic information and a relatively large endosperm containing most of the nutrient storage [7]. In mature rice endosperm, starch is a primary component with 80–90% of the total dry weight [8] and somewhat considered as a complex carbohydrate including amylose and amylopectin, which are synthesized and packed to form a large semicrystalline granule in amyloplasts through a large suite of enzyme activities [9]. Amylose is a linear chain made up of α-1,4 glycosidic linked glucose molecules with very few α-1,6 branches, whereas amylopectin, the main component of starch granule, is a highly branched chain of glucose units joined by both α-1,4 and α-1,6 glycosidic bonds [9,10,11]. Starch from different plant origins varies in its physicochemical properties due to the ratio of amylose and amylopectin and differences in branching density of semicrystalline structure [12]. Besides starch properties and functionalities, most studies of rice endosperm have been focused on starch biosynthesis protein expression and regulation [13,14,15]. Four main classes of starch biosynthesis enzymes including ADP-glucose pyrophosphorylase (AGPase), starch synthase (SS), starch branching enzyme (BE), and starch debranching enzyme (DBE) are presented in rice developing seeds [9,10,16].

Proteomics has become an important tool to study the dynamic and diverse biological processes and analyze expression patterns, variation, function, and interaction of proteins at a given time, in a particular tissue, or among different treatments of biotic and abiotic stresses in plants [16,17,18,19]. Using two-dimensional gel electrophoresis (2-DE)-based protein identification method equipped with mass spectrometric techniques, the profile of entire protein expression can be rapidly generated with highly reproducible [17,20], allowing for selection of the reasonable gene(s), which is possibly involved in regulatory mechanisms underlying starch biosynthesis [13,21,22]. In the last two decades, proteomic analyses of rice endosperm have been applied to a broad range of processes including differentially expressed proteins from the specific issues of plant tissues/organs [23], developmental stages [13,21,22,24,25,26], chalky and translucent parts [19], as well as under high temperature (HT) condition [13,27,28] in which the starch biosynthesis-related proteins were affected and reported.

The posttranslational modifications (PTMs) refer to the chemical modification events resulting from the covalent attachment of chemical groups, such as phosphate, acetyl, succinyl, methyl, and oligosaccharides, to amino acid side chains of the particular proteins [29]. PTM is a crucial step for functional protein maturation and important in signal transduction, apoptosis, transcriptional regulation, etc., by changing the chemical nature of polypeptide chains during or after protein biosynthesis [29,30]. Recently, a number of PTMs have been identified and verified by many highly effective techniques, coupled with database and bioinformatics tools. In rice endosperm, PTMs targeted starch biosynthesis proteins have been identified including phosphorylation [27,28,31], acetylation [30,32,33,34], succinylation [34], malonylation [35], and lysine 2-hydroxyisobutyrylation [36].

As the global population grows, it is expected that, by 2035, additional demand of approximately 112 million metric tons of rice needs to be produced to keep food security [37]. Understanding the function of proteomics and PTMs in rice seeds will contribute to our understanding of the mechanism and regulation of starch biosynthesis, which is one of the most important topics for high-yield and high-quality rice production [8,28,38,39]. This review summarizes the current knowledge in starch biosynthesis proteins through the studies of proteomics and PTMs, delving into the contribution of starch biosynthesis proteins to starch properties and functionality.

## 2. Significant Proteins for Starch Biosynthesis in Rice Seeds

### 2.1. Amylose and Amylopectin Biosynthesis

**Figure 2:**
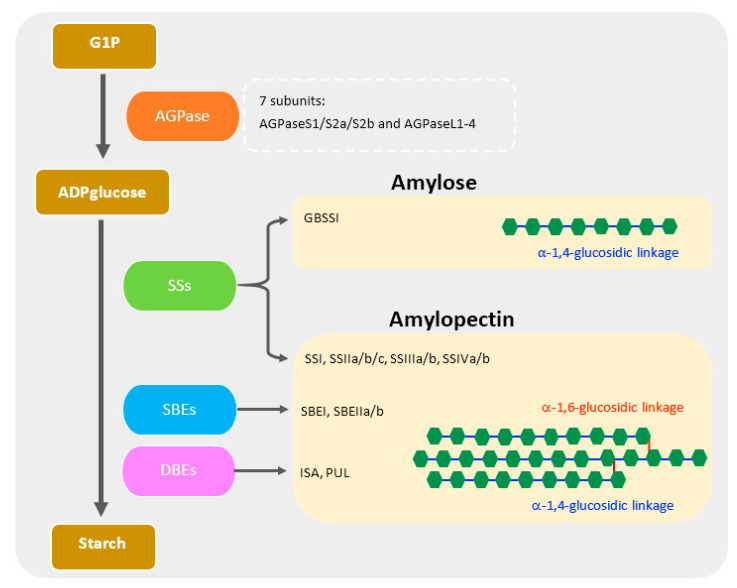
Brief schematic of the major proteins involved in starch biosynthesis in rice endosperm. Amylose and amylopectin are composed of glucose units (green rectangle) with α-1,4-glucosidic linkage (blue line). The branch chains of amylopectin are connected through α-1,6-glucosidic linkage (red line).

The enzymatic machinery for starch biosynthesis is summarized in Figure 2. AGPase (EC 2.7.7.27), a step limiting for starch biosynthesis [40,41], is a heterotetramer composed of large and small subunits, which catalyzes the glucose-1-phosphate (G1P) to ADPglucose [9,14,42,43]. In rice, seven AGPase subunits were reported [44]. To promote an α-1,4 bond and elongate the glucan chain, starch synthases (SSs) (EC 2.4.1.21) participate in the transfer of a glucosyl moiety from ADPglucose to the nonreducing end of the existing chains [45,46]. SSs are clustered into five classes including soluble SS I (SSI), SSII, SSIII, SSIV, and GBSS [45]. Two GBSS isoforms of rice—GBSSI and GBSSII—are primarily expressed in endosperm and leaves, respectively [44]. GBSSI is the only enzyme responsible for amylose biosynthesis (Figure 2).

In contrast, amylopectin, as compared with amylose, generally has a distinct fine structure and is synthesized by three main enzymes including SS, starch branching enzyme (BE), and starch debranching enzyme (DBE) [47]. The SSs play a crucial role in the elongation of α-1,4-glucosidic chains and also regulating the quality and quantity of starch, especially in defining amylopectin chain-length distribution [11,47]. In rice, SSs contain multiple paralogs including SSI, SSIIa(SSII-3)/IIb(SSII-2)/IIc(SSII-1), SSIIIa(SSIII-2)/IIIb(SSIII-1), and SSIVa(SSIV-1)/IVb(SSIV-2) [45]. Among those isoforms, SSI, SSIIa, and SSIIIa are responsible for priming the short degree of polymerization (DP) 6 to 7 chains [15], short A and B_1_ [42], and B_2_-B_4_ chains (DP ≥ 30) [43], respectively.

BEs (EC 2.4.1.18) specifically introduce the branch point, the α-1,6-glucosidic linkage into the glucan chain by cutting the α-1,4-linked glucan and also transferring to another chain at the 6-hydroxyl position and thus is considered as a key enzyme regulating amylopectin structure [48]. Three different isoforms of BEs including BEI, BEIIa, and BEIIb are present in rice endosperm [47]. BEI forms a variety of both short chains and intermediate chains (DP ≤ 40) by attacking the branched glucan in both the outer and inner chains of amylopectin [49]. Conversely, BEIIa and BEIIb function only in the outer amylopectin structure and specifically responsible for transferring the short chain of DP 6–15 and DP 6–7, respectively [47,49].

DBEs (EC 3.2.1.10) are composed of two types, isoamylase (ISA; EC 3.2.1.68) and pullulanase (PUL; EC 3.2.1.41), and hydrolyze the improper α-1,6-glucans [47]. Both ISA and PUL debranch the amylopectin as well as other substrates, i.e., glycogen and phytoglycogen, for ISA and pullulan for PUL [47]. However, the improperly located branches are mainly removed by ISA and partially by PUL [50].

Besides those main enzymes, starch phosphorylase (Pho; EC 2.4.1.1) also plays a crucial role in starch biosynthesis and degradation [51,52,53]. Pho catalyzes the phosphorolytic of the outermost glucose residue to generate G1P, which is reversibly added to the end of α-glucan chains [53], and recently, Pho is reported to play a crucial role in starch biosynthesis at low temperature [54]. Pho composted of a plastidial form or Pho1 (Pho-L) and a cytosolic form known as Pho2 (Pho-H) and both forms are different in terms of structure, kinetic properties, the expression pattern, and subcellular localization [53]. The enzyme activity of Pho1 is observed only in the endosperm of rice, while Pho2 is found in both endosperm and photosynthetic organs [53]. In addition, G1P is also the result of the reaction of plastidic phosphoglucomutase (PGM; EC 5.4.2.2), which catalyzes the conversion of G6P to G1P [55].

### 2.2. Phosphorylation and Dephosphorylation of Glucan Chains

Phosphorylation is only known in vivo covalent modification of starch. Two important isoforms of glucan water dikinase (GWD), GWD1 (EC 2.7.9.4) and phospho glucan water dikinase (PWD, EC 2.7.9.5) catalyze the phosphorylation at the C6 and C3 positions of glucosyl residues in amylopectin, respectively [56,57,58,59]. Both GWDs preferably phosphorylate the longer amylopectin chains at a range of DP 30–100 [57,60] and disrupt the glucan chains to access hydration [61]. The reduction of GWDs contributes to starch degradation by α-amylase (EC 3.2.1.1) [62]. In rice, *OsGWD1* (Os06g0498400) is responsible for leaf starch excess1 (LSE1) [63]. The LSE1 mutant lacked exons 22–32 displayed a higher starch content (5–10-fold) and lower phosphorylation (0.05 ± 0.02%) in the leaf blades, compared with those of wild type [63]. In addition, the LSE1 mutant had fewer panicles, lower ripened grains, smaller grains, and a lower grain yield, as compared to those of the wild type. Overexpression of potato *GWD1* in rice enhanced the phosphorylation at both C6 (9-fold) and C3 (2-fold) positions, increased amylose content, and displayed a minor change in starch granule morphology of endosperm [64]. Besides the alteration of starch properties and functionality [63,65,66], starch phosphorylation also interrupts the crystalline structure of amylopectin [67].

Dephosphorylation is an essential mechanism required for starch degradation [68]. Starch Excess4 (SEX4, EC 4.3.1.3.48) catalyzes the removal of phosphate groups at both C3 and C6 positions [68,69], while Like Sex Four2 (LSF2) specifically dephosphorylates at C3 position [69]. In Arabidopsis leaves, the deficiency of SEX4 caused an increase in starch accumulation [68,70], but a loss of LSF2 was found to have no obvious effects on starch levels [69]. Even though SEX4 and LSF2 play a crucial role in the dephosphorylation of Arabidopsis, their biological function remains unclear in rice endosperm. In addition, it was found that SEX4 was primarily expressed in the anthers of rice [71]. Recently, *OsSEX4* (LOC_Os03g01750/Os03g0107800)-knockdown rice caused an increase in starch accumulation in suspension-cultured cells, leaves, and rice straw, indicating that the function of *OsSEX4* is conserved with Arabidopsis [65]. The transgenic rice plants also exhibited a chalky grain phenotype and had no effects on vegetative growth and grain yield [65].

### 2.3. Disproportionation to Nonreducing End of Starch

The disproportionating enzyme (DPE, EC 2.4.1.25), a 4-α-glucanotransferase, cleaves the α-1,4 glucosidic bond, transfers the glucan moiety to the nonreducing end, and finally forms a new 1,4 glucosidic bond [66,72,73]. There are two isoforms of DPEs, plastid-located DPE1 and cytoplasm-located DPE2, which differ in expression profiles, subcellular localization, protein structure, and reaction properties [72,74]. The identified OsDPE1 and OsDPE2 are composed of 594 and 946 amino acids, respectively [72]. Both DPEs showed the conserved domain of glucoside hydrolase of family 77 (GH77). The OsDPE1 contains only one domain of GH77, while the OsDPE2 has two copies of GH77 at the C-terminal and two copies of N-terminal carbohydrate-binding module 20 (CBM20) [72]. The activities of DPEs are different; OsDPE1 catalyzes the maltotriose transfer reaction by using the glucose as its acceptor, whereas OsDPE2 participates in the glucose transfer reaction from maltose to glycogen acceptor [72]. Recently, DPE1 mediates the reaction of transferring maltooligosyl units from amylose and amylopectin to amylopectin [73].

### 2.4. Starch Granule Initiation

The initiation of starch granules is recently studied in order to understand the mechanisms and factors that influence the number of granules per plastid and the morphogenesis of granules [75]. Several key proteins playing a crucial role in granule initiation were discovered through the homologs of Protein Targeting to Starch (PTST) proteins [75,76]. PTST contains an N-terminal coiled-coil domain and a C-terminal carbohydrate-binding module 48 (CBM48) mediating protein–protein interaction and starch-binding domain, respectively [77,78]. In Arabidopsis chloroplast, PTST1 interacts directly with GBSS via the coiled-coil domain in the stroma and then locates to starch granules by using the CBM48 domain [78,79]. PTST2 interacts with SS4 and soluble maltooligosaccharides (MOS), while PTST3 interacts with PTST2 [80]. In addition, PTST2 is also associated with Mar-Binding Filament Protein (MFP1) to facilitate the normal PTST2 localization [81]. Therefore, the executive functions of PTST1, 2, and 3 are required for amylose biosynthesis, normal granule initiation, and cofunction with PTST2, respectively [80].

In rice, the functions of GBSS-binding protein (OsGBP) and Floury Endosperm6 (FLO6) are similar to PTST1 and PTST2, respectively. A newly identified OsGBP interacts directly with both GBSSI and GBSSII in yeast two-hybrid assay [82]. The coiled-coil domain is responsible for GBSS binding, while the CBM48 is essential for targeting GBSSs to starch granules during amylose biosynthesis [82]. Based on the CRISPR/Cas9 gene editing, mRNA and protein abundance of *osgbp* mutants were significantly decreased in both leaves and grains, compared to wild type, leading to the reduction of starch content and number of starch granules with smaller size in leave and the presence of large chalkiness area in the endosperm [82].

FLO6 plays an essential role in starch granule formation and contains N-terminal transit peptide and C-terminal CBM48 domain for plastid localization and binding to starch, respectively [83]. FLO6 interacts with ISA1 via its N-terminus with no effect on the enzyme activity. As compared to the wild type, the *flo6* mutant showed many smaller granules with irregular shapes and rough surfaces [83].

## 3. Proteomic Profiling of Starch Biosynthesis-Related Proteins

The proteome of each living cell refers to the entire proteins expressed by a genome, which is highly dynamic and altering in response to intra- and extracellular factors across time points [18]. With different purposes of proteomics analysis, proteins involving starch biosynthesis of rice endosperm have been intensively studied (Table 1, Table 2 and Table 3).

### 3.1. Specific Starch Biosynthesis-Related Proteins in Rice Seeds

To identify the tissue-specific expression in rice and understand the mechanisms that regulate starch biosynthesis, a total of 1022, 1350, and 877 unique proteins were identified from leaf, root, and seed tissues of rice, respectively, by using both 2-DE and high-performance liquid chromatography–tandem mass spectrometry (HPLC–MS/MS) coupled with multidimensional protein identification technology (MudPIT) [23]. The unique peptides of starch biosynthesis-related proteins were achieved for 7.43% (162/2180) of those from the root, followed by 2.29% (54/2358) and 0.37% (10/2712) from leaf and root tissues, respectively. Pho, AMY, ISA, and PGM were also observed in root tissue. AGPase and GBSS were detected in the leaf, while only PGM was observed in all three tissues (leaf, root, and seed) [23] (Table 1). At the mature stage of rice endosperm, 14 starch biosynthesis proteins were identified as the starch granule-associated proteins, and additional Hsp70, putative Brittle-1 protein, and PPDK were also identified (Table 1) [32].

Lin et al. [19] identified 113 differentially expressed proteins between the translucent and chalky parts of rice Wuyujing3. Among these, proteins in carbohydrate metabolism were the third most abundant (15.0%) after the categories of protein synthesis, folding and degradation (27.4%), and unidentified function (24.8%). AGPase, SSII, SSIII, SSIIIa, SBE, Pho1, PGM, and AMY were identified by using the isobaric tags for relative and absolute quantification (iTRAQ) based on the upper and the bottom half of translucent and chalky grains (Table 1). The AMY was downregulated in the chalky part, which was responsible for the processes of starch hydrolysis and chalk formation [19]. SSIIIa functions in B_2–4_ chains elongation with the degree of polymerization (DP) ≥ 30 [43]. In contrast, Lin et al. [19] found SSIIIa was increased in the chalky part, which was found the greater amount of short chain (DP ≤ 12) and fewer medium and long chains, compared with the translucent parts.

### 3.2. Starch Biosynthesis-Related Proteins in Different Developmental Stages of Rice Seeds

In cereal endosperm cells, starch granules are synthesized and increase in number and volume until maturity based upon the synergy of multiple enzymes [9,30,84,85]. No starch accumulation was observed in rice endosperm at 2 days after flowering (DAF), and a small amount of starch was found in the pericarp at 4 DAF [22]. A great accumulation of starch in endosperm was noticed after 8 DAF [22,86]. Endosperm remains equally milk white with no translucent region at 10 DAF [26] and 12 DAF [24]. Since the translucent region indicates the accumulation and packing of starch granule, half of the translucent area in the central endosperm were observed at 15 DAF [24,26], while full translucent in the whole endosperm was noticed at 20 DAF [26]. However, rice seed development varies depending on genotypic and environmental conditions [25].

The identified starch biosynthesis-related proteins involved in rice endosperm development were summarized in Table 2. Over 400 protein spots were identified in Taichung Native 1 (TN 1) at 12 DAF [13]. GBSS (Waxy) was identified and increased the expression after 6 DAF [13].

The 396 differentially expressed protein spots were identified in Nipponbare during the early (6–8 DAF), mid (8–12), and late (12–20) stages of rice seed development [22]. ISA I, AMY, DBE, Pho 1, PGM, and AGPase were increased from 6 to 20 DAF, while the expression level of ISA3 protein started to increase at 6 DAF, reached the highest level at 10 DAF, and decreased thereafter. During the late stage, the expression levels of PUL, Pho 1, and AGPase increased in abundance from 12 to 18 DAF [22].

Lee et al. [25] reported 4172 nonredundant proteins of fully mature seeds and 889, 913, 1095, and 899 proteins were identified during 10, 20, 30, and 45 DAF. Pho 1, PUL, AMY, SS 2–3, GWD, and SBE were differentially expressed among each interval stage and the highest protein abundance was observed at the fully mature grains (45 DAF). Among those proteins, PUL, AMY, and SBE increased until 20 DAF and slightly decreased at 30 DAF then rapidly increased at fully mature grain, suggesting that process of starch accumulation was intensive at 20 DAF [25].

Besides starch biosynthesis-related proteins, proteins involving in other metabolic processes such as glycolysis, TCA-cycle, lipid metabolism, and proteolysis were also detected at higher levels in the fully mature grain (desiccation phase), compared to the developing stages, suggesting that the accumulation of these proteins might be for seed germination [25].

Zhang et al. [87] found that AGPase, GBSS, and PUL were differentially expressed between superior and inferior spikelets during the rice grain-filling stage, which was downregulated in the inferior spikelets at the early stage. Western blotting indicated that AGPase was downregulated in the inferior spikelets at all stages of the early, mid, and late grain-filling stage, compared to superior spikelets [87]. In addition, SBE 3, AGPase, PUL, and SBE 1 were detected as the interacting proteins with 14–3-3 [88], which might play a crucial role in the termination of inferior spikelets’ development.

Yu et al. [26] identified 115 developmentally changed starch granule-associated proteins (SGAPs), with 39% of which involving in starch biosynthesis. Pho1, PUL, SSI, and AGPase S2a slowly increased in abundance from 10 to 15 DAF and then rapidly increased from 15 to 20 DAF, while the levels of GBSSI abundance showed the linearly decreased from 10 to 20 DAF [26].

Overall, the expression patterns of proteins involving in starch biosynthesis, starch degradation, and starch phosphorylation are particularly responsible for rice seed development. Most of those reported proteins are continuously increased during 6–20 DAF. However, proteins involving in starch debranching, degradation (AMY, Pho1, and PUL), and phosphorylation (GWD) are markedly decreased at 30 DAF and then increased to complete seed development.

### 3.3. Starch Biosynthesis-Related Proteins Respond to High Temperature (HT)

HT particularly affects the yield and quality of rice [89] but no significant changes were observed for seed morphology and size [90]. The effects of HT on starch biosynthesis-related proteins were summarized in Table 3.

HT at 35/30 °C (day/night) decreased the GBSS abundance of japonica rice (TNG 67), leading to the lower amylose content observed at 15 DAF (12.3 ± 0.5%), compared with the control temperature at 30/25 °C (15.6 ± 0.4%), while those of indica rice (TN1) were relatively stable under both temperatures [13]. According to the HT condition during grain filling, chalkiness occurrence is increased, and loosely packed of abnormal starch granules were observed in rice endosperm [92,93]. Li et al. [91] reported that the conditions of both day high temperature (35/27 °C, DHT) and night high temperature (27/35 °C, NHT) caused a higher percentage of chalkiness but lower levels of brown rice rate, milled rice rate, head rice rate, amylose content, and gel consistency, compared to the control condition (28/20 °C). Besides a PGM isoform, five isoforms of PUL were detected and differentially accumulated in response to DHT and NHT [91]. Compared to heat-sensitive rice (XN0437S), the lower abundance of PUL, DBE, and GBSS was observed in heat-tolerant rice (XN0437T) at 1 day (d), 3 d, and 5 d of HT stress (38.0 ± 0.5 °C) [89]. The higher accumulation of AGPase L was found in heat-tolerant rice at 1 d and decreased at 3 d and 5 d of HT stress [89].

Compared to the perfect grain, SSI, SSII, PUL, GBSSI, and BEIIb were downregulated, while AMY (AmyII-3) was increased in chalky grain in both moderate (24.4 °C) and HT (28.0 °C) conditions [92]. DBE and BEI were down- and increased in moderate and HT conditions, respectively [92].

Moreover, Timabud et al. [90] reported that heat stress affected the abundance of starch biosynthesis proteins in milky, dough, and mature stages. Under heat treatment, SBE3, AGPase L2, SSIIa, and SSI were expressed and detected only at the milky stage, while SBEI and GBSSI were increased in abundance from milky to the dough but not detectable at the mature stage. AMY was highly expressed at the milky, compared to the dough stage, whereas AGPase was increased in the highest abundance at the mature stage. AGPase S2 was differentially expressed in both dough and mature stages, while ISA was detected at the milky and mature stages [90]. In addition, grains weight under HT were increased more rapidly than the control, especially from late milky to middle dough stages [90,91].

Altogether, HT activates starch degradation rather than starch biosynthesis through the reduction of GBSSI, SSI, SSII, SBEIIb, DBE, and PUL, while some proteins, such as AGPase L, GBSSI, SBEI, and AMY are increased and contributed to the lower amylose content and the higher the chalkiness rate in rice endosperm.

## 4. Starch Biosynthesis-Related Proteins Targeted by PTMs

**Figure 3:**
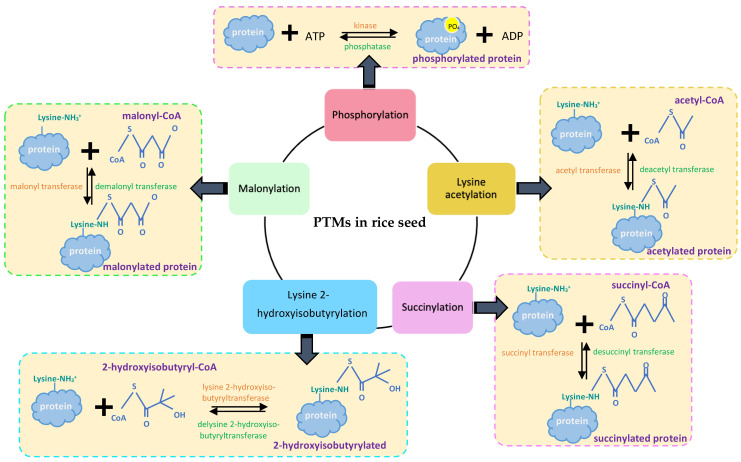
Five types of PTMs targeting starch biosynthesis proteins identified from rice seeds.

**Figure 4:**
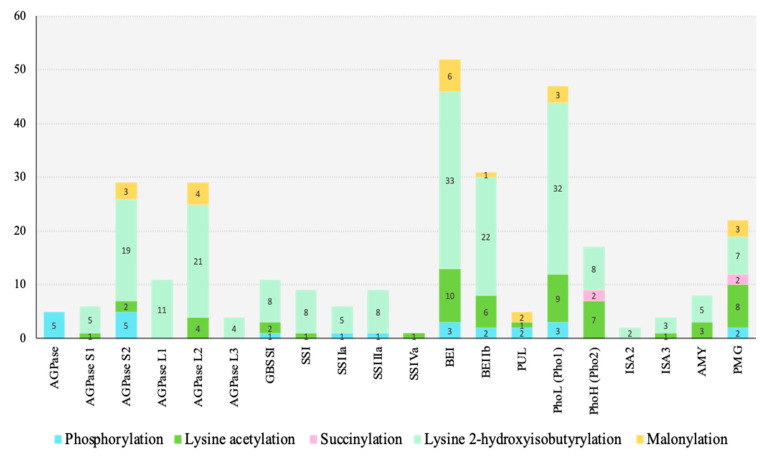
Summary of PTMs targeting starch biosynthesis-related proteins in rice seeds. The number on stacked bar indicates total target sites of PTM(s) identified for each protein.

**Figure 5:**
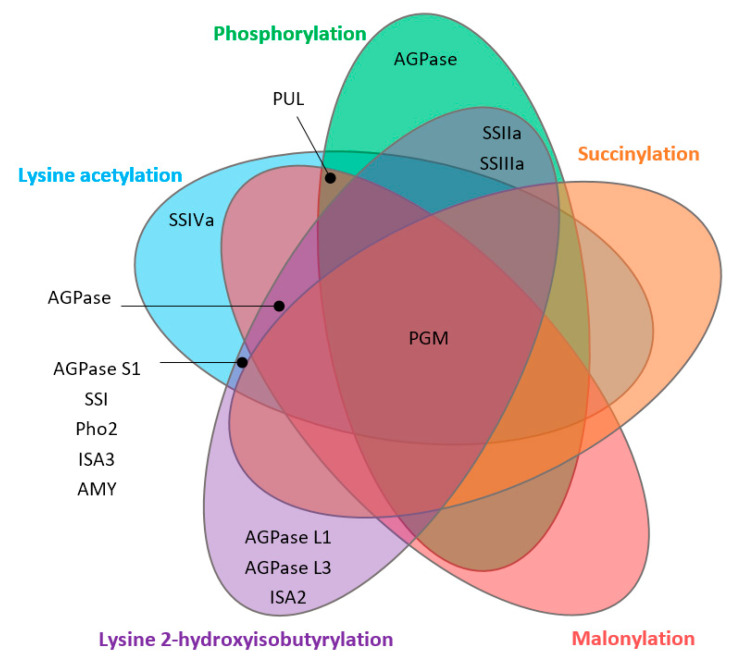
Venn diagram displays the overlap of starch biosynthesis-related proteins targeted by five types of PTMs identified from rice seeds.

PTMs can alter structure formation, activity, stability, structure, and localization of proteins, which are necessary for cellular functions [84,85]. Five types of PTMs targeting starch biosynthesis proteins have been reported from rice seeds (Figure 3, Figure 4 and Figure 5).

### 4.1. Phosphorylation

#### 4.1.1. Identification of Phosphorylated Protein in Rice Developing Seeds

Protein phosphorylation is a reversible process regulated by kinases and phosphatases and regarded as one of the most important PTMs [94]. The studies on phosphorylation have mainly focused on phosphorylation and/or dephosphorylation of specific proteins or protein families in particular signaling pathways [95]. In eukaryotes, the most common class of phosphorylation is found on serine (S), threonine (T), and tyrosine (Y) residues [96,97] in which the γ-phosphate group covalently reacts with the hydroxyl group of amino acid side chains by potein kinases [98]. A large scale of phosphosites and phosphoproteins has been identified using phosphoproteomic technologies in many cereals such as rice [27,28,31,99,100,101,102,103], wheat [104,105,106,107,108], barley [109,110], and maize [111,112,113,114,115]. Phosphoserine (>90%) is a major phosphorylation type of rice endosperm, followed by phosphothreonine (6–9%) and phosphotyrosine (0.1–0.4%) [28,31]. Nakagami et al. [116] estimated that the proportion of phosphotyrosine in rice is equivalent to that in Arabidopsis and humans.

Recently, the phosphoproteins involved in starch biosynthesis of indica rice cultivars (9311 and Guangluai4) including AGPase (three sites) AGPS2 (five sites), SSIIa (one site), SSIIIa (one site), BEI (four sites), BEIIb (two sites), PUL (three sites) and Pho1 (two sites) were reported by Pang et al. [28] (Table 4). 

Interestingly, one phosphopeptide of both AGPase and SSIIIa showed consistency between japonica and indica rice [28,31]. AGPase and GBSS were detected as downregulated phosphoproteins during grain-filling stages of inferior spikelets, as compared to superior spikelets [87]. Moreover, PGM and AGPase were differentially phosphorylated between the superior and inferior spikelets in which the expression levels of those phosphoproteins of 10 DAF inferior spikelets were lower than both 1 DAF superior spikelets and 20 DAF inferior spikelets [88].

#### 4.1.2. Potential Role of Protein Phosphorylation in Starch Biosynthesis

In amyloplasts, starch biosynthesis isozymes have been demonstrated to display as a complex form (or protein–protein interactions), especially through the regulation of phosphorylation [117,118,119,120]. In wheat (*Triticum aestivum*), phosphorylation can activate SBEIIa and SBEIIb enzymes contributed to the protein complex forming of SBEIIa, SBEIIb, and Pho1 at 12–25 days after pollination (DAP) [117]. On the other hand, dephosphorylation reduces the catalytic activities and breaks the complex formation [117]. Furthermore, the phosphorylation-dependent complexes of wheat SSI, SSIIa, and either SBEIIa or SBEIIb were identified in amyloplast at 10–15 DAP [119].

In maize (*Zea mays* L.) endosperm, SSI, SSIIa, and SBEIIb form a trimeric complex in which SBEIIb is phosphorylated [121]. The complex formation is activated by ATP and disassembled by alkaline phosphatase [118,121]. Loss of SBEIIb activity (*amylose extender*, *ae*^−^ mutant) impacts the protein–protein interactions among SSI, SSIIa, and SBEIIb complex, which is formed in wild type [118]. It was reported that SSI and SSIIa formed the complex possibly through SBEI, SBEIIa, and Pho in the *ae*^−^ mutant. Since the SBEIIb is replaced by SBEI, a reduction of branch points with longer glucan chains was observed in *ae*^−^ mutant, as compared to the wild type [118,121]. Recently, the phosphorylated SSIIa is reported that to have interactions with SSI and SBEIIb [120]. In addition, in barley (*Hordeum vulgare*), protein complex formation of SBEIIa, SBEIIb, SSIIa, and SSIIIa was increased by the presence of ATP [122].

In rice endosperm, phosphorylation and dephosphorylation affected the oligomerization and activity of OsGBSSI [123]. The dissociation of OsGBSSI was detected during the phosphatase treatment. The monomer of OsGBSSI increased from 0.07 to 0.86%, and the OsGBSSI activity decreased from 0.17 to 0.11 mol/g/min based upon the increasing phosphatase levels [123]. In addition, GBSSI expression at low temperatures was regulated by phosphorylation [124].

Protein complex formations among starch biosynthesis proteins have been established in rice including the interactions of SSs-SBEs, among SBE isoforms, BEIIa-Pho1, PUL-BEI [125,126,127], PUL-BEIIb [126], Pho1-Dpe1 [128], and SSI-SSIIa-BEIIb [126]. Recently, an inactive BEIIb forming a complex with SSI, SSIIa, SSIVb, BEI, and BEIIa was reported [126]. Although the evidence of phosphorylation-dependent complex assembly in rice has not been uncovered, the enzymatic activities and enzymatic complexes in rice might be regulated by phosphorylation, as reported in other cereals.

Taken together, phosphorylation is essential for the regulation of starch biosynthesis and has significant effects on enzymatic activities, complex components, and protein–protein interactions. Knockout of one enzyme may lead to changes in protein complex formation, other enzyme activities, and amylopectin structure.

### 4.2. Lysine Acetylation

Lysine acetylation, a highly conserved PTM in organisms, is well known for the regulation of transcription [129] and reported in a large number of proteins in many biological processes of organisms [130]. Lysine acetylation is a reversible reaction in which an acetyl group (CH_3_Co) from acetyl–coenzyme A (CoA) is donated to N^ε^-terminal amine of a lysine residue through acetyltransferases and removed by deacetylases [131]. The acetylation controls the enzymatic activities of metabolic enzymes and alters the metabolic flux profiles [132]. In rice seeds, starch biosynthesis proteins targeted by lysine acetylation were listed in Table 5.

In total, 2, 2, 2, 10, 2, and 8 sites of lysine acetylation modifications identified from starch granules of mature rice endosperm were reported for AGPase S2, AGPase L2, GBSSI, SBEI, SBEIIb, and Pho L, respectively [32] (Table 5). PGM, Pho L, and AMY were acetylated with six, four, and one sites at the early stage of rice seed germination (24 h after imbibition) [34]. In addition, Wang et al. [30] reported the largest acetylome based on three different developing stages of rice seeds (unpollinated pistil, 3 DAP, and 7 DAP) covering 1817 acetylsites from 972 acetylproteins. Seven starch biosynthesis proteins were targeted by acetyllysine including AGPase S1 (one site), AGPase S2 (two sites), AGPase L2 (three sites), AMY (four sites), SBE3 (three sites), Pho L (two sites), and SSI (one site) (Table 5).

Compared to root, leave, flower, and pollen, the prominent acetylated proteins of rice were observed in seeds at 7, 15, and 21 DAF, as well as in mature dry seeds [33]. A total of 1003 acetylated sites on 692 proteins were identified from rice seeds at 15 DAF [33]. Interestingly, 11 starch biosynthesis proteins were lysine-acetylated including AGPasae S2, AGPasae L2, GBSSI, SSIVa, SBEI, SBEIIb, ISA3, PUL, PMG, Pho H, and Pho L (Table 5).

Among those acetylated proteins, SBEI and Pho L were heavily acetylated on 10 and 8 sites, respectively, which were in a similar result of starch granule acetylome [32]. Out of 7 distinguished motifs of acetylation site reported by Meng et al. [33], 6 motifs displayed amongst the 11 acetylated starch biosynthesis proteins including KF (5), KY (4), KXXXR (3), KXXXXR (1), KXXXK (4) and KH (4) (Table 5).

Lysine acetylation has a strong impact on the biochemical functions of proteins [133]. For example, Ribulose-15-bisphosphate carboxylase/oxygenase (Rubisco) activity was decreased by lysine acetylation [134], the increased glyceraldehyde-3-phosphate dehydrogenases (GAPDH) acetylation in leaves of *Brachypodium distachyon* L. enhanced the activity in glycolysis and decreased the activity in gluconeogenesis [135]. On the other hand, de-acetylation can increase the pyruvate orthophosphate dikinase (PPDK) activity in maize after 12 h of illumination with white light [134]. It is plausible that lysine acetylation may influence the catalytic activities of starch biosynthesis proteins in rice.

### 4.3. Succinylation

Succinylation is recently identified as one of the PTMs on lysine residue [136,137] and plays an important role in gene transcription, cellular metabolism, DNA damage response [138], and plant growth [135]. Zhang et al. [136] reported that succinyl–CoA plays a role as a cofactor for lysine succinylation. Among 261 acetylated proteins identified in rice embryos at 24 h after imbibition, two sites from both PGM (8 and 18) and Pho H (412 and 439) were succinylated, which might be involved in the metabolism regulation [34]. Lysine residue at 18 positions on PGM (Uniprot: Q9AUQ4) was targeted for both acetylation and succinylation modifications (Table 5).

Previous studies indicated that succinylation has a potential impact on protein structure and functions [136] as well as cellular processes [139]. Succinylation alters the electrophoretic mobility and isoionic pH of ovalbumin [140], inactivates the canonical carnitine palmitoyltransferase (CPTase) activity, decreases the enolase activity [141], and promotes cell invasion and migration [139]. Succinylation in plants has been reported in rice [34], tomato [142], *B. distachyon* L. [135], patchouli [143], and tea [144]. However, the effect of lysine succinylation in plants is relatively limited.

### 4.4. Lysine 2-Hydroxyisobutyrylation (K_hib_) and Malonylation (K_mal_)

K_hib_ is a highly dynamic PTM found on both histone and nonhistone proteins affecting the histone–DNA association and playing role in diverse biological processes [36,145]. K_hib_ introduces a huge change in size, as compared to lysine acetylation, and particularly forms hydrogen bonds with other molecules via its hydroxyl group [145].

In contrast, K_mal_, a lately identified lysine acylation, is evolutionarily conserved in mammalian and bacteria cells [146,147] and responsible for the regulation of cellular mechanisms and activities [35,146]. K_mal_ triggers more dramatic structural changes than both lysine acetylation and methylation on the substrate proteins [147]. Recently, both K_hib_ and K_mal_ were identified from the developing rice seeds at 15 DAF reported by Meng et al. [36] and Mujahid [35], respectively (Table 6).

A total of 2512 K_hib_ proteins were reported in rice [36] and amongst those proteins, 17 proteins involving in starch biosynthesis were targeted (Table 6). The highest modified sites of K_hib_ were observed on SBEI (33 sites), followed by Pho L (32 sites) and AGPase L2 (21 sites). The K_hib_ function on targeted proteins remains unknown in rice. However, there was evidence that K_hib_ modification may introduce the conformational changes of Enolase1 and alter the substrate binding [148]. K_hib_ increased the hydrophobic solvent-accessible surface area and decreased the enzymatic activity of UvSlt2 [149].

For K_mal_, seven starch biosynthesis proteins out of 247 malonylated proteins were reported [35]. The number of the modified sites of AGPase S2, AGPase L2, SBEI, SBEIIb, Pho L, PUL, and PGM was 3, 4, 6, 1, 3, 2, 3, respectively (Table 6). Although malonylation can occur on several enzymes of the starch biosynthesis mechanism, the potential roles of K_mal_ remain largely unknown. The malonylation has an important role in the enzymatic activity of various proteins. For example, the enzymatic activity of malonylated fructose bisphosphate aldolase B (ALDOB) was decreased by 20%, as compared to nonmalonylated ALDOB [150]. Malonylation increased the enzymatic activity of glyceraldehyde-3-phosphate dehydrogenase (GAPDH) and also interrupted its binding to the target mRNAs [151]. Moreover, cells with elevated K_mal_ had the impaired mitochondrial function and fatty acid oxidation [152].

## 5. Summary and Future Perspectives

Even though identification of the proteomics and PTMs has been conducted in rice developing seeds, there still remain significant gaps in their regulations in starch biosynthesis. This review presents the significant proteins associated with starch biosynthesis in rice seeds and their expression profiles through different developmental stages and high temperatures. Most of the key proteins in starch biosynthesis are generally increased in the endosperms during 6–20 DAF. SSIIIa and AMY are promising proteins in chalky formation. Hight temperature initiates starch degradation rather than starch biosynthesis and then also results in the reduction of amylose content as well as the increase in chalkiness in rice seeds. Twenty starch biosynthesis proteins are targeted by five types of PTMs including phosphorylation, lysine acetylation, succinylation, lysine 2-hydroxyisobutyrylation, and malonylation. PGM is commonly targeted by all the five PTMs types (Figure 5). Phosphorylation is the most important PTM for starch biosynthesis proteins regarding the regulation of protein complex formation. This information is useful to understand the molecular mechanisms underlying starch biosynthesis, which ultimately affect starch functionality with known and unknown regulatory pathways. Further studies, however, may be focused on the following aspects:

(1) Proteome alteration under climate change environment: Recently, the global population is facing challenging problems caused by global warming and climate change, which have a great impact on rice yield and quality. Further studies are needed to determine the consequences of climate change, e.g., high/low temperatures, carbon dioxide levels, drought stress, etc., on starch biosynthesis mechanism and regulation by using proteomic analysis.
(2) The number and new types of PTMs in rice seeds: Although five types of PTMs were identified from rice seeds, whether there are other PTMs in rice seed has not been fully addressed. For the number of PTMs sites, K_hib_ showed the highest number of targeted starch biosynthesis proteins (17 proteins), while the lowest number was observed in succinylation (2 proteins). Whether more PTMs would be found under the specific genotype or under the specific abiotic conditions such as heat stress, high carbon dioxide levels, etc., is unknown.
(3) The roles and regulation mechanisms of PTMs on starch biosynthesis: Little is known about the roles of individual PTM on the starch biosynthesis proteins and the impact of PTMs on enzymes’ activities, protein–protein interaction (protein complex formation), and starch functionality. The phosphorylation is well reported in protein complex formation during the starch biosynthesis process in the endosperm of cereal crops. In-depth regulatory studies on protein–protein interactions are necessary to understand the role of protein complex formation in starch biosynthesis in different crops.

## Figures and Tables

**Table 1:** List of starch biosynthesis proteins of rice endosperm based on proteomics in response to the organ-/tissue-specific differences.

Sample	Aim of Study	Technique	Identified Proteins	Details of Results
leaf, root, and seed of Nipponbare (*Japonica*) [23]	To identify protein expression in leaf, root (49 DAG), and seed (14 DAG).	2-DE HPLC–MS/MS MudPIT	AGPase (id: 7670) leaf, seed AGPase small subunit (id: 44074) leaf, seed AGPase (id: 9904, 34550) seed AGPase (id: 50182) leaf GBSS (id: 31122) leaf, seed GBSS (id: 31130) seed SS precursor (id: 99443, 52528) seed SS (id: 26269) seed SBE isoform rbe3 (id: 36892) seed SBE (id: 20648, 27094, 53238, 55740) seed DBE (id: 14376) PhoH isoenzyme (id: 12500) seed Pho1 (id: 32714) seed AMY precursor (id: 21708) seed AMY (id: 24707) seed ISA (id: 23091) seed ISA (id: 23496) seed PGM, chloroplast precursor (id: 34039) seed PGM, cytoplasmic (id: 38302) leaf, root, seed	Proteins involving in starch biosynthesis were observed in both leaf and seed tissues. Starch degradation-related proteins were observed only in seed tissue. Two isoforms of small AGPase subunit were detected in both leaf and seed whereas another two isoforms of large AGPase subunit were identified only in seed tissue. The third isoform of large AGPase subunit was observed in leaf.
DY1102 (Wuyujing3 (*Japonica*) treated with 0.5% ethyl methanesulfonate (EMS)) (notched-belly mutant with white belly) [19]	To identify the differentially expressed proteins between the chalky and the translucent parts of DY1102 grains.	iTRAQ LC-MS/MS	AGPase, SSII, SSIII, SBE, Pho1, PGM, AMY, and putative starch synthase DULL1 (SSIIIa)	Downregulation of AMY was observed in chalky part. Downregulation of AMY contributes to starch hydrolysis and the formation of chalkiness.
				SSIIIa was one of the differentially expressed proteins and increased in chalky part. The increase in SSIIIa expression did not result in the increased proportion of long amylopectin chains (DP > 30).
Nipponbare (*Japonica*) [32]	To develop a method for rice starch granule purification from mature endosperm and identify starch granule-associated proteins.	LC-MS/MS	AGPase S2, AGPase L1, AGPase L2 GBSSI, GBSSII SBE 1, SBE3 SSI, SSII-1, SSII-3, SSIIIa PUL Pho1 ISA2	Besides 14 identified starch biosynthesis proteins, the other candidate starch granule-associated proteins involving in starch biosynthesis were also identified by LC-MS/MS including Hsp70, putative Brittle-1 protein, and PPDK. Compared with Tris-HCl buffer extraction method, the proteome extracted by the phenol buffer had more proteins and displayed almost all identified proteins extracted by Tris-HCl buffer.

**Table 2:** List of starch biosynthesis proteins of rice endosperm based on proteomics in response to different developmental stages.

Sample	Aim of Study	Technique	Identified Proteins	Details of Results
Taichung Native 1 (TN 1, *Indica*) and Tainung 67, (TNG 67, *Japonica*) [13]	To investigate the changes in protein expression patterns during rice caryopsis development (6, 9, 12, 15, and 32 DAF).	2-DE LC-MS/MS	GBSS (Waxy)	The expression of GBSS increased after 6 DAF was coincident with the increase in amylose content. GBSS protein was highly expressed in kernels of rice with high amylose content (TN1).
Nipponbare (*Japonica*) [22]	To study the protein expression profiles related to grain filling during 6–20 DAF.	2-DE MALDI-TOF/TOF	ISA I, AMY, Pho, PGM, AGPaseL2, AGPaseL3, AGPaseS2a/b	All identified proteins were continuously increased from 6 to 20 DAF. Some AGPase isoforms had the highest peak of protein expression at 16 DAF and decrease thereafter.
			ISA3	ISA3 increased at 6 DAF, showed the highest expression at 10 DAF, and decreased thereafter.
			SSI	No result of expression pattern.
Zhonghua 10 (*Japonica*) [24]	To study the cellular features and proteomics of rice endosperm from 12, 15, and 18 DAF.	2D-DIGE MALDI-TOF/ TOF-MS	PUL Pho1 AGPase L AGPase S2	Most of the protein expression patterns showed increase in abundance from 12 to 18 DAF. Some isoforms of PUL and AGPase S2 had the highest peak of expression at 15 DAF. Pho1 decreased the expression level form 12–18 DAF. AGPase L showed the highest variation of expression patterns including the expression levels continuously decreased and increased from 12–18 DAF, showed the highest peak and lowest peak at 15 DAF. The completion of starch granule packing was firstly observed in the inner part of endosperm at 15 DAF and showed entire endosperm at 18 DAF. AGPase L and Pho1 were significantly coexpressed with proteins in redox regulation (SOD and APX, respectively)
Ilpumbyeo (*Japonica*) [25]	To identify the differentially expressed proteins of rice grains at 10, 20, 30 DAF and the fully mature grain (45 DAF).	MudPIT	Pho1 PUL AMY SS 2–3 GWD SBE	All identified 6 starch biosynthesis proteins were reproducibly identified and differentially expressed during four stages (10, 20, 30, and 45 DAF). All of these proteins had the highest expression levels at the fully mature grain except SS 2–3 in which its abundance increased until 20 DAF after that decreased at 30 DAF and increased at fully mature grain. The authors suggested that the expression profile of starch biosynthesis proteins was similar to previous research of Xu et al. [22]
Jinhui No. 809 (*Indica*) [87]	To identify the differentially expressed proteins between superior (SS) and inferior spikelet (IS) at the early (EGS), mid (MGS), and late (LGS) grain-filling stages.	2-DE MALDI-TOF/MS LC-ESI-MS/MS	AGPase GBSS PUL	AGPase, GBSS, and PUL isoforms were downregulated in inferior spikelets at EGS.
			AGPase S	AGPase S showed downregulation in both MGS and LGS.
Jinhui No. 809 (*Indica*) [88]	To identify the differentially expressed proteins of 10 DAF superior spikelet (SS) and 10 and 20 DAF inferior spikelet (IS).	2-DE MALDI-TOF/MS LC-ESI-MS/MS	AGPase GBSS SBE 1 SBE 3 PUL	AGPase had lower expression level in 10 DAF IS compared with both 10 DAF SS and 20 DAF IS. SBE 3, AGPase, PUL, and SBE 1 were detected as the 14-3-3 interacting proteins. AGPase and GBSS might be involved in the developmental stagnancy stage (DSS) of IS especially at the early grain-filling stage.
Zhonghua 10 (*Japonica*) [26]	To identify the SGAPs of rice at 10, 15, and 20 DAF.	2D-DIGE MALDI-TOF/ TOF-MS	Pho1 PUL SSI AGPase L2 GBSSI AGPase S2a	Protein abundance of Pho1, PUL, SSI, and AGPase S2a slowly increased from 10 to 15 DAF and then drastically increased from 15 to 20 DAF. GBSSI showed linearly decreased abundance levels from 10 to 20 DAF. GBSSI and SSI were found only in starch granule-associated (SGA) form. AGPase, Pho1, and PUL were observed in both soluble and SGA forms.

**Table 3:** List of starch biosynthesis-related proteins of rice endosperm based on proteomics in response to HT.

Sample	Aim of Study	Technique	Identified Proteins	Details of Results
Taichung Native 1 (TN 1, *Indica*) and Tainung 67, (TNG 67, *Japonica*) [13]	To determine the candidate proteins associated with grain quality under HT, 35/30 °C (day/night).	2-DE LC-MS/MS	GBSS (Waxy)	HT caused the reduction of GBSS in TGN67 and decreased the levels of amylose content of TNG67 at 15 DAF (12.3 ± 0.5%) compared with those (15.6 ± 0.4%) under the control temperature (30/25 °C). Protein expression of TN1 showed relatively stable in both HT and control conditions. TNG67 showed more sensitivity to HT than TN1.
9311 (*Indica*) [91]	To identify the differentially accumulated proteins of rice at 5, 10, 15, and 20 DAF under day HT (DHT, 35/27 °C) and night HT (NHT, 27/35 °C).	2-DE MALDI-TOF MS/MS	PGM PUL	One and five isoforms of PUL and PGM were differentially accumulated in response to DHT and NHT and detected in all 5, 10, 15, and 20 DAF with different accumulation patterns. Three PUL isoforms (spot 34, 35, and 36) were increased in parallel abundance from 5 to 20 DAF, while another (spot 37 and 38) showed slowly increase at 5–10 DAF and highly increase in abundance at 15 and 20 DAF.
XN0437T (heat-tolerant) XN0437S (heat-sensitive) [89]	To identify the differentially expressed proteins during rice grain development at 1, 3, and 5 day after HT treatment (38.0 ± 0.5 °C) compared with control (25.0 ± 0.5 °C)	2-DE MALDI-TOF/TOF MS	PUL DBE GBSS AGPase L	All 4 proteins involving in starch biosynthesis showed downregulation in both rice lines under HT stress compared with the control treatment. AGPase L was higher accumulated in the heat-tolerant rice at 1 day after HT and showed lower accumulation at 3 and 5 days after HT compared to heat-sensitive rice. PUL, DBE, and GBSS had lower expression levels in heat-tolerant rice at all three-time points.
Perfect and chalky rice grains (Koshihikari (*Japonica*)) [92]	To study the proteomic profile of the translucent and opaque grains under moderate (in 2009, 24.4 °C) and HT (in 2010, 28.0 °C) conditions.	iTRAQ MS/MS	SSI SSII PUL GBSSI BEIIb	All identified proteins showed downregulation in chalky rice compared to perfect grain. Protein expression of SSII, PUL, and BEIIb under moderate temperature was lower than HT condition, while the others, SSI and GBSSI showed higher abundance under HT.
			AMY (AmyII-3)	AMY showed upregulation in chalky rice in both conditions and chalky rice under HT stress had higher AMY abundance than moderate temperature.
			DBE BEI	Both DBE and BEI were increased in HT but downregulated in moderate temperature.
KDML105 (*Indica*) [90]	To identify the differentially changed proteins of rice grains under heat stress (40/26 °C) at the milky, dough, and mature stages.	nanoLC-MS/MS	AMY	AMY showed the highest in abundance at milky then decreased in dough and disappeared at mature stage.
			AGPase	AGPase had the un-change expression in both milky and dough stages and double increased in mature stage.
			SBEI GBSSI	Protein abundance of both SBEI and GBSSI was increased almost three times from milky to dough stages. Both SBEI and GBSSI were not found at mature stage.
			AGPase L2	AGPase L2 was detected only at dough stage.
			SBE3 AGPase L2 SSIIa SSI	All proteins were detected only in milky stage in which the AGPase L2 showed the highest abundance followed by SSI, SBE3, and SS IIa.
			ISA	ISA was detected in both milky and mature stages and showed the highest abundance at mature stage.

**Table 4:** List of identified phosphoproteins involving in starch biosynthesis in rice endosperm based on phosphoproteomics.

Phosphorylated Protein	Uniprot ACCN	Identified Phosphosite(s) ^a^	Subspecies	Reference
AGPase	B8XEC3	S62, S381	*indica*	[28]
		T68	*japonica and indica*	[28,31]
	A2Y7W1	S491	*japonica*	[27]
	-	-	*indica*	[87,88]
AGPS2	D4AIA3	S13	*japonica and indica*	[28,31]
		S17, S22, S35, S36	*Indica*	[28]
GBSSI	-	-	*indica*	[87]
SSIIa	P0C586	S126	*indica*	[28]
SSIIIa	Q6Z1D6	S96	*japonica and indica*	[28,31]
BEI	D0TZI4	S562, S620, S814, S815	*indica*	[28]
BEIIb	A2X5K0	S685, S715	*indica*	[28]
PUL	D0TZH1	S154, S155, S869	*indica*	[28]
Pho1	Q9AUV8	S494, S645	*indica*	[28]
PGM	Q9AUQ4	S124	*japonica*	[31]
	Q33AE4	S167	*japonica*	[31]
	-	-	*indica*	[88]

^a^ S and T indicate the phosphorylated site on serine and threonine residues, respectively.

**Table 5:** List of lysine acetylation and succinylation on starch biosynthesis proteins from rice seeds.

Acetylated Protein	Uniprot ACCN	Acetylation Position	Modified Peptide ^a^	Lysine Motif ^b^	Tissue-Specific ^b^	Reference
AGPase S1	Q69T99	203	MDYQK(ac)FIQAHR	-	-	[30]
AGPase S2	P15280	217	MDYEK(ac)FIQAHR	-	starch granule/seeds (7 DAP)/seeds (15 DAF)	[32]/[30]/[33]
		261	IVEFAEK(ac)PK	KF	starch granule/seeds (unpollinated pistil and 7 DAP)/seeds (15 DAF)	[32]/[30]/[33]
AGPase L2	Q5VNT5	250	ASDYGLVK(ac)FDDSGR	KF	starch granule/seeds (3 and 7 DAP)/seeds (15 DAF)	[32]/[30]/[33]
		260	VIAFSEK(ac)PK	-	starch granule	[32]
		310	DVLLDILK(ac)SK	-	Seeds (7 DAP)	[30]
		312	SK(ac)YAHLQDFGSEILPR	-	Seeds (7 DAP)	[30]
GBSSI	Q0DEV5	444	KFEK(ac)LLK	-	starch granule/seeds (15 DAF)	[32]/[33]
		452	SMEEK(ac)YPGK	KY	starch granule/seeds (15 DAF)	[32]/[33]
SSI	Q0DEC8	193	NFANAFYTEK(ac)HIK	-	seeds (3 and 7 DAP)	[30]
SSIVa	Q5JMA0	589	AQYYGEHDDFK(ac)R	-	seeds (15 DAF)	[33]
SBEI	Q0D9D0	89	LEEFK(ac)DHFNYR	-	starch granule/seeds (15 DAF)	[32]/[33]
		103	YLDQK(ac)CLIEK	-	starch granule/seeds (15 DAF)	[32]/[33]
		118	HEGGLEEFSK(ac)GYLK	KXXXK	starch granule/seeds (15 DAF)	[32]/[33]
		164	DK(ac)FGIWSIK	KF	starch granule/seeds (15 DAF)	[32]/[33]
		236	YVFK(ac)HPR	KH	starch granule/seeds (15 DAF)	[32]/[33]
		372	GYHK(ac)LWDSR	KXXXXR	starch granule/seeds (15 DAF)	[32]/[33]
		614	EGNNWSYDK(ac)CR	-	starch granule/seeds (15 DAF)	[32]/[33]
		662	QIVSDMNEK(ac)DK	-	starch granule/seeds (15 DAF)	[32]/[33]
		697	VGCDLPGK(ac)YR	KY	starch granule/seeds (15 DAF)	[32]/[33]
		809	GM(ox)K(ac)FVFR	KXXXR	starch granule/seeds (15 DAF)	[32]/[33]
SBEIIb	Q6H6P8	134	VVEELAAEQK(ac)PR	-	seeds (15 DAF)	[33]
		303	YIFK(ac)HPQPK	KH	Seed (7 DAP)/seeds (15 DAF)	[30]/[33]
		587	WSEK(ac)CVTYAESHDQALVGDK	-	seeds (unpollinated pistil and 7 DAP)	[30]
		688	FIPGNNNSYDK(ac)CR	-	seeds (7 DAP)	[30]
		738	KHEEDK(ac)MIIFEK	-	starch granule/seeds (15 DAF)	[32]/[33]
		771	VGCLKPGK(ac)YK	KY	starch granule/seeds (15 DAF)	[32]/[33]
ISA3	Q6K4A4	130	K(ac)YFGVAEEK	KY	seeds (15 DAF)	[33]
PUL	Q7X834	805	NEENWHLIK(ac)PR	-	seeds (15 DAF)	[33]
PMG	Q9AUQ4	8	VLFSVTK(su)K	-	embryos (24 HAI)	[34]
		18	ATTPFDGQK(ac)PGTSGLR	-	embryos (24 HAI)/seeds (15 DAF)	[34]/[33]
			ATTPFDGQK(su)PGTSGLR		embryos (24 HAI)	[34]
		69	YFSK(ac)DAVQIITK	-	embryos (24 HAI)	[34]
		206	LMK(ac)TIFDFESIK	-	embryos (24 HAI)	[34]
		215	TIFDFESIK(ac)K	-	seeds (15 DAF)	[33]
		275	EDFGGGHPDPNLTYAK(ac)ELVDR	-	embryos (24 HAI)	[34]
		361	NLNLK(ac)FFEVPTGWK	-	embryos (24 HAI)	[34]
		506	DPVDGSVSK(ac)HQGVR	KH	embryos (24 HAI)/seeds (15 DAF)	[34]/[33]
		543	VYIEQYEK(ac)DSSK	KXXXK	seeds (15 DAF)	[33]
PhoH	Q8LQ33	169	YGLFK(ac)QCITK	-	embryos (24 HAI)	[34]
		409	HMEIIEEIDK(ac)R	-	embryos (24 HAI)	[34]
		412	FK(su)EMVISTR	-	embryos (24 HAI)	[34]
		439	ILDNSNPQK(su)PVVR	-	embryos (24 HAI)	[34]
		645	LVNDVGAVVNNDPDVNK(ac)YLK	-	embryos (24 HAI)	[34]
		747	FEEAK(ac)QLIR	KXXXR	seeds (15 DAF)	[33]
		818	MSILNTAGSGK(ac)FSSDR	-	embryos (24 HAI)	[34]
PhoL	Q9AUV8	216	YK(ac)HGLFK	KH	starch granule/seeds (unpollinated pistil, 3 DAP and 7 DAP)/seeds (15 DAF)	[32]/[30]/[33]
		255	TDVSYPVK(ac)FYGK	KXXXK	starch granule/seeds (15 DAF)	[32]/[33]
		451	YGTEDTSLLK(ac)K	-	starch granule/seeds (15 DAF)	[32]/[33]
		504	SLEPSVVVEEK(ac)TVSK	KXXXK	starch granule/seeds (15 DAF)	[32]/[33]
		594	FQNK(ac)TNGVTPR	-	starch granule/seeds (15 DAF)	[32]/[33]
		734	AFATYVQAK(ac)R	-	seeds (7 DAP)	[30]
		846	AQGK(ac)FVPDPR	KF	starch granule/seeds (15 DAF)	[32]/[33]
		913	DQK(ac)LWTR	KXXXR	starch granule/seeds (15 DAF)	[32]/[33]
		928	MSILNTASSSK(ac)FNSDR	KF	starch granule/seeds (15 DAF)	[32]
AMY	Q0J528	88	LYDLDASK(ac)YGTEAELK	-	embryos (24 HAI)/-	[34]
		123	CADYK(ac)DSR	-	-	[30]
	P27933	88	LYDLDASK(ac)YGTAAELK	-	-	[30]
		215	GYSTDIAK(ac)MYVESCK	-	-	[30]

^a^ (ac) and (su) indicate the acetylation and succinylation sites on lysine, respectively. ^b^ K is the position of the acetylated or succinylated lysine, and X refers to a random amino acid residue.

**Table 6:** Summary of lysine 2-hydroxyisobutyrylation (K_hib_) and malonylation (K_mal_) on starch biosynthesis proteins from 15 DAF developing rice seeds identified by Meng et al. [36] and Mujahid et al. [35], respectively.

Protein	Uniprot ACCN	No. of K_hib_ and K_mal_ *	Position
AGPase S1	Q69T99	5	203, 234, 249, 442, 462
AGPase S2	P15280	19	102, 132, 217, 239, 248, 261, 263, 268, 285, 360, 385, 403, 406, 441, 447, 456, 467, 476, 496
		3 *	106, 360, 403
AGPase L1	Q6AVT2	11	100, 194, 196, 247, 299, 331, 326, 369, 446, 456, 470,
AGPase L2	Q5VNT5	21	37, 74, 187, 223, 250, 263, 273, 286, 301, 302, 310, 312, 334, 364, 371, 392, 425, 443, 449, 459, 504,
		4 *	250, 312, 371, 449
AGPase L3	Q688T8	4	202, 228, 315, 376
GBSSI	Q0DEV5	8	181, 192, 309, 381, 385, 530, 538, 549
SSI	Q0DEC8	8	193, 196, 349, 357, 429, 461, 467, 570
SSII-3	Q0DDE3	5	151, 244, 346, 378, 532
SSIIIa	Q6Z1D6	8	228, 649, 761, 794, 808, 961, 1203, 1604
SBEI	Q0D9D0	33	62, 64, 84, 89, 103, 108, 118, 122, 157, 164, 171, 186, 215, 236, 319, 324, 372, 423, 500, 506, 524, 540, 549, 614, 662, 664, 683, 689, 697, 744, 775, 796, 809
		6 *	108, 118, 506, 524, 689, 809
SBEIIb	Q6H6P8	22	134, 146, 158, 191, 231, 268, 299, 328, 386, 466, 558, 564, 571, 587, 603, 612, 636, 677, 688, 719, 738, 773,
		1 *	719
AMY	Q0J528	5	39, 88, 105, 207, 262
	Q0JJV2	1	88
ISA2	Q6AU80	2	319, 369
ISA3	Q6K4A4	2	266, 269
PhoH	Q8LQ33	8	115, 409, 425, 533, 542, 595, 721, 818,
PhoL	Q9AUV8	32	134, 255, 259, 277, 289, 356, 381, 410, 418, 429, 441, 451, 471, 493, 504, 590, 617, 630, 636, 657, 665, 681, 725, 734, 738, 846, 893, 904, 913, 928, 940, 946,
		3 *	259, 493, 657
PUL	Q7X834	2 *	274, 871
PGM	Q33AE4	7	61, 67, 118, 413, 492, 584, 595
	Q9AUQ4	3 *	54, 458, 568

* Indicates the number of K_mal_.

## Data Availability

All data presented in this review can be found in the references cited in the text.
